# Kite Versus Ponseti Method in the Treatment of Idiopathic Congenital Clubfoot: A Systematic Review and Meta-Analysis

**DOI:** 10.7759/cureus.63030

**Published:** 2024-06-24

**Authors:** Elshymaa E Raslan, Basel H Bakhamees, Leenah A Turjoman, Noor N Alalqam, Batool N Alalqam, Bahja J Alhaddad, Abdallah Alim, Asma M Alharbi, Ali H Alqahtani, Olaa M Omaish, Batoul AlEdwani, Rawyah Dawas

**Affiliations:** 1 Surgery, King Khalid Hospital, Tabuk, SAU; 2 Faculty of Medicine, King Abdulaziz University, Jeddah, SAU; 3 Surgery, Almaarefa University, Riyadh, SAU; 4 Faculty of Medicine, Mansoura University, Mansoura, EGY; 5 Ophthalmology, Salmaniya Medical Complex, Manama, BHR; 6 College of Medicine, King Khalid University, Abha, SAU; 7 College of Medicine, Princess Nourah Bint Abdulrahman University, Riyadh, SAU; 8 Surgery, University of Tabuk, Tabuk, SAU; 9 College of Medicine, King Saud Bin Abdulaziz University for Health Sciences, Jeddah, SAU

**Keywords:** relapse, ponseti method, meta-analysis, kite method, correction, clubfoot

## Abstract

Kite and Ponseti methods are two popular manipulating methods for correcting the deformity of idiopathic congenital clubfoot. We aimed to compare the efficacy of Kite and Ponseti methods in the treatment of children with idiopathic congenital clubfoot. A search was launched on Medline/PubMed, Cochrane Central Register of Controlled Trials, the Web of Science, ProQuest, and Scopus without limits, from inception to May 1, 2024. The outcomes included the rates of initial correction and relapse (primary) as well as the number of casts and duration of treatment (secondary). Mean difference (MD) and risk ratio (RR) were calculated for numerical and dichotomous outcomes, respectively, with 95% confidence intervals (CIs). Nine studies were included. Meta-analysis showed the Ponseti method is significantly associated with a higher probability of correction (n = 6, RR = 1.23 [95% CI = 1.14, 1.32], p < 0.001) and a lower risk of relapse (n = 5, RR = 0.50 [95% CI = 0.36, 0.71], p < 0.001) compared to the Kite method. The Ponseti method utilized a lower number of casts (MD = -3.0 [95% CI = -5.8, -0.2], p = 0.04) and took a shorter duration (MD = -39.76 [95% CI = -67.22, -12.30], p = 0.02) than the Kite method. Evidence suggests that the Ponseti method results in better outcomes than the Kite method in terms of successful initial correction and lower relapse rates. However, the available studies showed varying degrees of risk of bias, and the length of follow-up was inadequate in some studies.

## Introduction and background

Idiopathic clubfoot, also known as talipes equinovarus, is a congenital deformity that affects the mid and forefoot in children. The deformity involves the following components: cavus, equinus, varus, and adductus [1]. Clubfoot is not spontaneously corrected with foot growth. Its prevalence ranges from 1 to 2 per 1,000 live births, making it the most common deformity in children [2]. The condition presents commonly in isolation (idiopathic clubfoot) or as part of a syndrome such as arthrogryposis multiplex congenita or spina bifida [3].

The development of clubfoot entails the interaction of several environmental and genetic factors [4]. The pathogenesis of clubfoot is attributed to the excessive formation of collagen, which leads to fibrosis and the shortening of ligaments, tendons, and muscles in the feet [5]. In the absence of treatment, clubfoot results in rigidity of the affected limb and leads to fixed alteration in gait [6].

At present, the first line of treatment is conservative manipulation and casting [7,8]. Several conservative methods have been developed for correcting deformities, with the Ponseti and Kite methods being the most popular [9,10].

The Kite method was developed in the 1930s and aims to achieve a sequential and gradual correction of each deformity. The method starts with the correction of forefoot adduction, followed by the correction of the inversion deformity of the hindfoot varus. Finally, the ankle equinus deformity is addressed. The correction of each deformity is initiated only after the previous deformity has been fully corrected in the aforementioned sequence [11]. Kite reported a 90% success rate for this technique. Nevertheless, several studies failed to reproduce the high rates of success observed in Kite’s series [12]. In addition, further surgical treatment was required for 20-50% of patients after the Kite method [12].

In the 1950s, Ponseti developed his technique, but it became only widely used in the 1990s [13,14]. The Ponseti technique corrects the midfoot cavus, hindfoot varus, and forefoot adduction simultaneously, while the equinus deformity is treated later. In selected cases, percutaneous Achilles tendon tenotomy is performed to enhance the correction of equinus deformity [13]. Several studies have reported a 90% success rate for the Ponseti method [9,15,16]. However, lower success rates were obtained by other studies, with higher rates of relapse or the need for surgical treatment [17]. This meta-analysis aimed to compare the efficacy of the Kite and Ponseti methods in the treatment of children with idiopathic congenital clubfoot.

## Review

Methodology

The meta-analysis followed the principles of the Cochrane Handbook for Systematic Reviews of Interventions, Version 6. The methods and results were reported following the Preferred Reporting Items for Systematic Reviews and Meta-Analyses (PRISMA) guidelines [18].

Eligibility Criteria for the Included Studies

Types of studies: This meta-analysis included English-language randomized and non-randomized clinical trials as well as cohort studies without time restrictions.

Participants: Studies were included if patients were diagnosed with idiopathic congenital clubfoot.

Interventions: Direct comparison between the Ponseti method and the Kite method.

Exclusion criteria: Conference abstracts, duplicate reports, case reports, review articles, editorials, clinical guidelines, and studies that assessed one method only (single-arm studies) were excluded.

Search Strategy

The search was launched in the electronic databases of Medline/PubMed, Cochrane Central Register of Controlled Trials (CENTRAL), Web of Science, ProQuest, and Scopus. No filters were used. The search included all studies from inception till May 1, 2024. The search terms included “clubfoot” AND “Ponseti” AND “Kite.”

Selection of Studies

Two independent reviewers performed the search, screened the obtained search results, and revised the full text of apparently eligible studies. Disagreements between the two reviewers were settled by consensus.

Data Extraction

The extracted data included (a) the study design, period, eligibility criteria, sample size, and follow-up; (b) patients’ age at starting treatment and sex; and (c) the outcomes, i.e., number of casts, duration of treatment, number of corrected and relapsed feet, Pirani scores.

Measured Outcomes

Primary outcome: Comparison of the risk of successful initial correction at the end of treatment and the risk of relapse at the end of follow-up between the two groups.

Secondary outcomes: Comparison of the number of casts and the duration of applying the method of treatment between the two groups.

Assessment of the Risk of Bias in Included Studies

The risk of bias (ROB) was assessed using the ROB2 tool for randomized clinical trials (RCTs) and the MINORS checklist for non-randomized and observational studies [19,20]. The ROB2 tool consists of five domains that assess the randomization process, deviations from the assigned treatment, missing data, measurement of the outcome, and selective reporting of the outcomes and results. In addition, an overall ROB can be assessed from the five domains. The MINORS checklist consists of 12 questions that are assigned points from 0 to 2. The overall ROB was assessed by summing the score and dividing it into tertiles, with the first, second, and third tertiles indicating high, uncertain, and low risk, respectively [21].

Data Synthesis

Analyses were conducted using the R Statistical language (version 4.3.3) [22] using the packages meta (version 7.0.0) [23], and dmetar (version 0.1.0) [24]. Numerical outcomes were presented using the mean difference (MD) by subtracting the mean of the Ponseti group from the mean of the Kite group. Dichotomous outcomes were presented using the risk ratio (RR). Significant heterogeneity was identified if the p-value from the Cochrane chi-square test was below 0.1 and the I^2^ index was 50% or above. The results were pooled using a fixed-effect model if heterogeneity was non-significant; otherwise, the random-effects model was used [25]. A p-value <0.05 was selected for interpreting the comparisons between the two arms. We did not perform testing for publication bias because the number of included studies was less than 10.

Results

Results of the Literature Search and Study Selection

**Figure 1:**
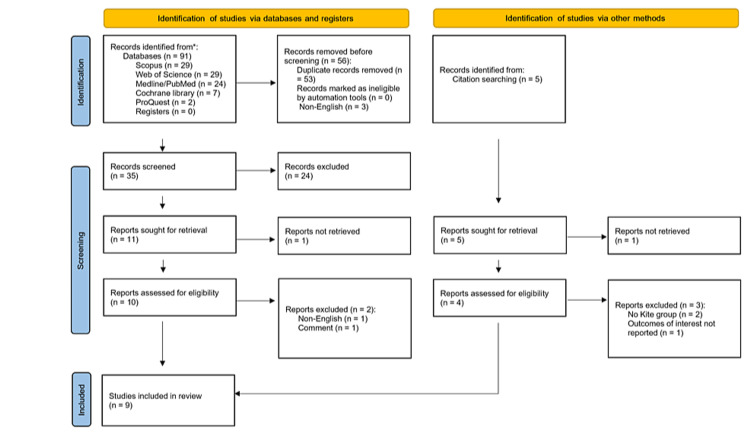
Preferred Reporting Items for Systematic Reviews and Meta-Analyses flowchart diagram for the results of the literature search and study selection.

The online search of databases returned 91 records. Of these, 53 records were duplicates which were removed while three records were available only in non-English languages. The remaining 35 records were screened by reviewing their titles and abstracts, with the exclusion of 24 records. The full text of one article was not available [26]. The full texts of the remaining 10 records were retrieved and evaluated for the eligibility criteria of the meta-analysis. We excluded two records: one was a comment on an article [27], and the other lacked full English text [28], leaving eight records for inclusion [12,16,29-34]. Screening of citations retrieved five records, of which the full text of one record was not retrieved [35], two lacked a Kite group [36,37], and one record did not report the outcomes of interest [38]. Overall, nine studies were finally included in this systematic review (Figure 1) [12,16,29-34,39].

Basic Characteristics and Assessment of the Risk of Bias of the Included Studies

Table 1 and Table 2 outline the main characteristics of the nine included studies. Five studies were RCTs [12,16,29,30,33], while two studies were retrospective cohorts [32,34], and one study was quasi-experimental [39]. The duration of follow-up was above one year in four studies only (Table 1) [12,16,29,34]. The participants’ age, sex, and laterality of the clubfeet as well as the studies’ inclusion and exclusion criteria are listed in (Table 2).

**Table 1:** Characteristics of the included studies (n = 9). NR = not recorded; RCT = randomized controlled trial

Study	Study design	Study period	Sample size Ponseti:Kite	Follow-up (months)
Sud et al. (2008) [12]	RCT	From March 2003 through February 2004	23:22 patients 36:31 feet	Mean ± SD = 27.2 ± 3.1 Mean ± SD = 24.8 ± 3.5
Sanghvi and Mittal (2009) [29]	RCT	NR	21:21 patients 30:34 feet	Mean ± SD = 36 ± 4
Rijal et al. (2010) [30]	RCT	Between July 2005 and May 2006	26:24 patients 30:30 feet	2.5
Selmani (2012) [16]	RCT	From January 2006 through February 2009	50:50 patients 76:74 feet	Mean ± SD = 36.2 ± 3.2 Mean ± SD = 35.1 ± 2.5
Kaseke and Mudawarima (2013) [31]	Prospective non-randomized study	From March 2011 to August 2011	14:11 patients 20:18 feet	1.5
Derzsi et al. (2015) [32]	Retrospective cohort	Between January 2007 and 2013	106:129 feet	6
Garcia et al. (2018) [33]	RCT	From January 2012 to May 2013	50:50 patients 71:55 feet	NR
Chen et al. (2019) [34]	Retrospective cohort	From 2003 to 2008	19:38 patients 30:58 feet	Mean (range) = 70 (51–84) Mean (range) = 73 (58–96)
Sharif et al. (2021) [39]	Prospective non-randomized study	NR	22:24 patients 30:30 feet	2.5

**Table 2:** Patients’ characteristics and eligibility criteria of the included studies (n = 9). Bil = bilateral; F = female; Lt = left; M = male; NR = not recorded; Rt = right; SD = standard deviation

Study	Groups	Age (days), mean ± SD	Gender (M/F)	Clubfoot laterality Rt/Lt/Bil	Inclusion criteria	Exclusion criteria
Sud et al. (2008) [12]	Ponseti	31.8 ± 27.4 days	14/9	4/6/13	Classical idiopathic clubfeet; age <3 months	Age >3 months; non-idiopathic deformities
	Kite	26.1 ± 21.4 days	17/5	5/8/9		
Sanghvi and Mittal (2009) [29]	Ponseti	NR	13/8	6/6/9	Idiopathic clubfeet	Coexisting myelocele, meningomyelocele, arthrogryposis multiplex congenital, or other neuromuscular disorders
	Kite		14/7	5/3/13		
Rijal et al. (2010) [30]	Ponseti	195.7 ± 202.8 days	76.2% males	-	Idiopathic congenital clubfoot	Age >2 years; prior surgical intervention
	Kite			-		
Selmani (2012) [16]	Ponseti	35.3 ± 25.4 days	30/20	26 bilateral	Classical idiopathic clubfeet; age <3 months	Coexisting myelocele, meningomyelocele, arthrogryposis multiplex congenital, and other neuromuscular disorders; age >3 months; or non-idiopathic deformities
	Kite	32.5 ± 26.3 days	28/22	24 Bilateral		
Kaseke and Mudawarima (2013) [31]	Ponseti	<12 months	17/8	6 bilateral	Age <1 year; idiopathic congenital clubfoot; no prior treatment	Prior treatment; coexisting disorder (e.g., myelodysplastic, arthrogrypotic, or other neuromuscular disorders)
	Kite			7 bilateral		
Derzsi et al. (2015) [32]	Ponseti	5.2 ± 2.0 days	93/68	97/64/74	Idiopathic clubfeet presenting within the first week of life	Not complying with treatment and aftercare; abandoning the therapy; refusing to participate
	Kite	5.2 ± 1.6 days				
Garcia et al. (2018) [33]	Ponseti	10 days to 12 months	58/42	21 bilateral	Idiopathic congenital clubfoot; age ≤12 months	Non-idiopathic deformity; coexisting pathology (e.g., myelodysplastic, neurological, or arthrogrypotic disorders)
	Kite			5 bilateral		
Chen et al. (2019) [34]	Ponseti	12.3 ± 7.0 days	12/7	3/5/11	Classical idiopathic clubfeet; age <3 months	Other congenital deformities, syndromic, or neurological causes of clubfeet
	Kite	14.4 ± 8.3 days	26/12	7/11/20		
Sharif et al. (2021) [39]	Ponseti	10.8 ± 4.6 weeks	12/10	11/3/8	Idiopathic clubfoot; age <6 months	NR

The ROB was assessed using the ROB2 tool for RCTs (Table 3) and the MINORS checklist for observational and non-randomized studies (Table 4).

**Table 3:** The risk of bias assessment for the included randomized clinical trials based on the ROB2 tool (n = 5). ROB = Risk of Bias

Study	Randomization process	Deviations from intended interventions	Missing outcome data	Measurement of the outcome	Selection of the reported result	Overall
Sud et al. (2008) [12]	High	High	High	Low	Some concerns	High
Sanghvi and Mittal (2009) [29]	Some concerns	Some concerns	Low	Low	Some concerns	Some concerns
Rijal et al. (2010) [30]	Some concerns	Some concerns	Low	Low	Some concerns	Some concerns
Selmani 2012 [16]	Some concerns	High	High	Low	Some concerns	High
Garcia et al. (2018) [33]	High	High	Low	Low	Some concerns	High

**Table 4:** The risk of bias assessment for the included non-randomized and observational studies based on the MINORS checklist (n = 4). Q1: A clearly stated aim; Q2: Inclusion of consecutive patients; Q3: Prospective collection of data; Q4: Endpoints appropriate to the aim of the study; Q5: Unbiased assessment of the study endpoint; Q6: Follow-up period appropriate to the aim of the study; Q7: Loss to follow up less than 5%; Q8: Prospective calculation of the study size; Q9: An adequate control group; Q10: Contemporary groups; Q11: Baseline equivalence of groups; Q12: Adequate statistical analyses; the items are scored 0 (not reported), 1 (reported but inadequate), or 2 (reported and adequate). ROB = risk of bias; MINORS = Methodological Items for Non-randomized Studies

Studies	Q1	Q2	Q3	Q4	Q5	Q6	Q7	Q8	Q9	Q10	Q11	Q12	Total	Overall ROB
Kaseke and Mudawarima (2013) [31]	2	0	0	2	0	0	0	1	2	2	0	1	10	Uncertain
Derzsi et al. (2015) [32]	2	2	0	1	0	0	0	0	2	2	1	1	11	Uncertain
Chen et al. (2019) [34]	2	2	0	2	0	2	0	0	2	2	2	1	15	Uncertain
Sharif et al. (2021) [39]	2	0	1	2	0	0	0	0	2	2	2	1	12	Uncertain

Regarding the RCTs, the domains of randomization, deviation from intended intervention, and selective reporting showed some concerns or high ROB in all studies. Two studies did not report the details of randomization [12,33]¸ while allocation concealment was uncertain in all five studies. In addition, baseline characteristics were not compared between the two groups in the study by Sanghvi et al., whereas the study by Garcia et al. showed a considerable difference in the rate of bilateral clubfeet between the two groups [29,33]. The domain of deviations from intended outcomes showed a lack of information about the blinding of participants’ families [12,16,29,30,33], and intention-to-treat analysis was not used or not clearly stated [12,16,33]. Selective reporting showed some concerns as all studies did not have a published protocol to compare the outcomes and methods of analysis to those already performed in the study. A high ROB arose from missing data in the studies by Sud et al. and Selmani (Table 3) [12,16].

The items that showed high ROB in the observational studies included unclarity regarding the inclusion of consecutive patients [31,39], collection of data [31,32,34], unclarity about the blinding of outcome assessors and loss to follow-up >5% in all four studies, duration of follow-up [31,32,39], no prior sample size calculation [32,34,39], and baseline equivalence of groups (Table 4) [31].

Results of Meta-Analysis

**Figure 2:**
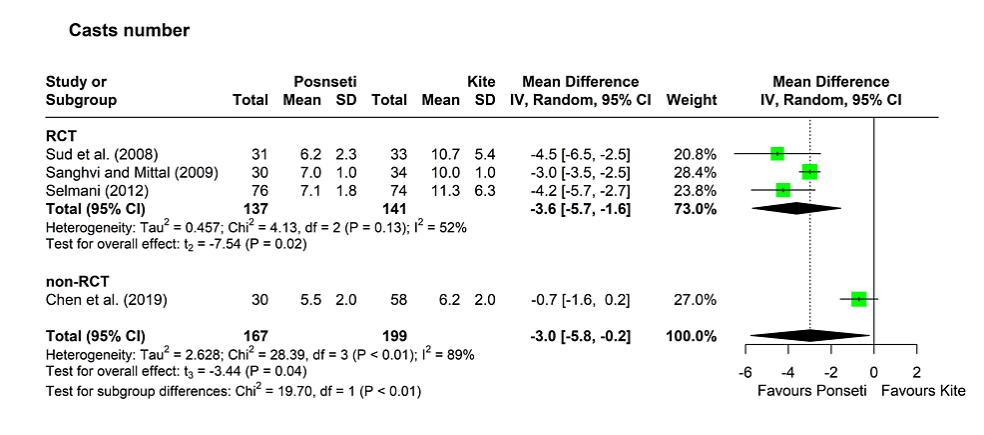
Forest plot showing pooling of the studies’ findings regarding the number of casts. References: [12,29,16,34]. CI = confidence interval; MD = mean difference

Number of casts: Four studies reported a lower mean number of casts in the Ponseti group compared to the Kite group [12,16,29,34], with significant differences in three studies [12,16,29]. There was considerable heterogeneity among the studies (chi-square = 28.39, p < 0.01, I^2^ = 89%), so pooling of the results was achieved using the random-effects model. The pooled MD [95% confidence interval (CI)] was -3.0 [-5.8, -0.2], with a p-value of 0.04. The leave-one-out analysis identified the study by Chen et al. as an outlier and its removal decreased the I^2^ index to 52% [34]. On assessing the three RCTs, the pooled MD indicated a significant decrease in the number of casts in the Ponseti group (MD [95% CI] = -3.6 [-5.7, -1.6], p = 0.02) (Figure 2).

**Figure 3:**
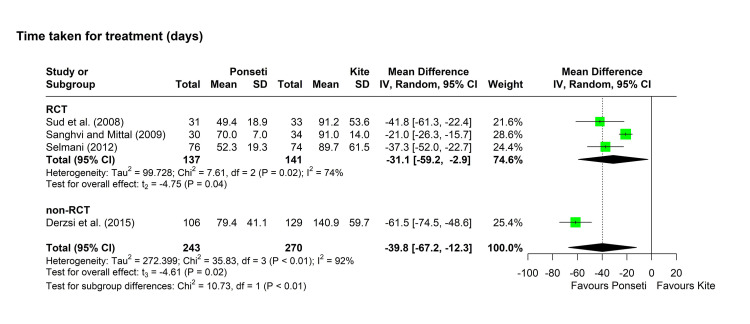
Forest plot showing pooling of the studies’ findings regarding the time taken for casting. References: [12,29,16,32]. CI = confidence interval; MD = mean difference

Time taken (days): Four studies compared the time required for applying the methods between the two groups, with a significantly lower mean duration in the Ponseti group compared to the Kite group [12,16,29,32]. Pooling of results was performed using the random-effects model due to the marked heterogeneity among the studies (chi-square = 35.83, p < 0.001, I^2^ = 92%). The pooled MD [95% CI] was -39.76 [-67.22, -12.30] days, with a p-value of 0.02 (Figure 3). The leave-one-out analysis identified the study by Derzsi et al. as an outlier and its removal decreased the I^2^ index to 74% [32]. On assessing the three RCTs after the removal of the outlier which was a non-RCT study, the pooled MD indicated a significant decrease in the number of casts in the Ponseti group (MD [95% CI] = -31.09 [-59.24, -2.94], p = 0.04) (Figure 3).

Total Pirani score: Three studies compared Pirani score measurements between the two groups at various time points [30,31,39]. The study by Rijal et al. assigned a subset of patients with bilateral clubfeet so that one foot was treated with one method and the other foot was treated with the other method [30]. They found that the mean Pirani scores were significantly lower in the legs treated by the Ponseti method compared to the other legs treated by the Kite method, and this significant difference was noticed from the third week and continued till the tenth week. In the remaining participants (unilateral and bilateral clubfeet, but patients were assigned to one group only), there was more reduction in Pirani score in the Ponseti group, but statistical significance was noticed in the ninth and tenth weeks only.

The study by Kaseke and Mudawarima reported that the mean Pirani scores were significantly lower in the Ponseti group compared to the Kite group at three and six weeks [31]. The study by Sharif et al. showed also significantly lower Pirani scores in the Ponseti group, starting from the fourth week and still detected in the tenth week [39].

**Figure 4:**
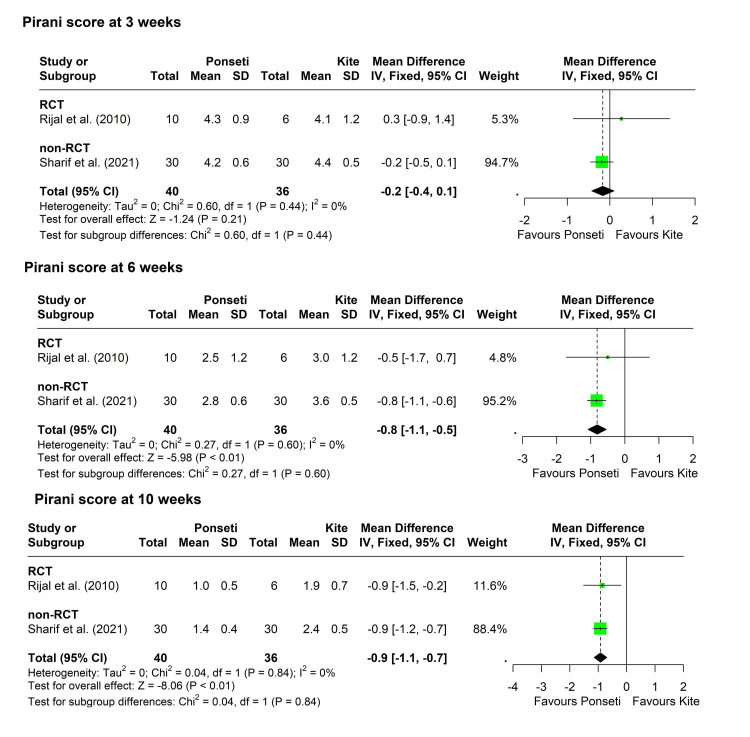
Forest plot showing pooling of the studies’ findings regarding Pirani scores at three, six, and ten weeks. References: [30,39]. CI = confidence interval; MD = mean difference

Pooling of the results showed that the MD between the two groups indicated lower scores in the Ponseti group at the third, sixth, and tenth weeks, with statistically significant results at the sixth and tenth weeks. The fixed-effect model was used at the three time points due to negligible heterogeneity. Only two studies were included in the meta-analysis, as the study by Kaseke and Mudawarima did not report enough data for inclusion in the analysis [31]. The results of unilateral legs were used from the study by Rijal et al. (Figure 4) [30].

**Figure 5:**
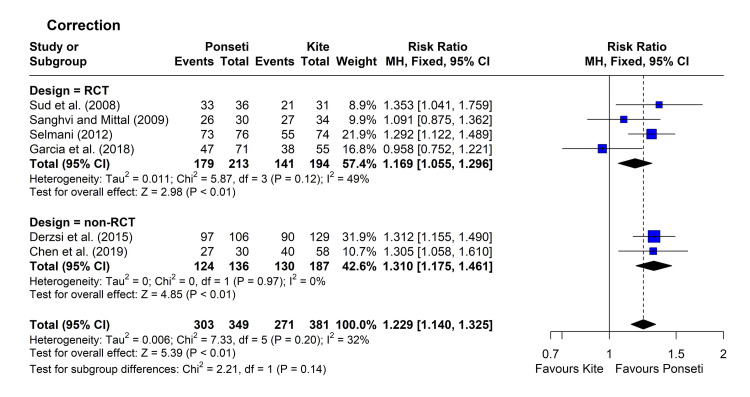
Forest plot showing pooling of the studies’ findings regarding the rate of successful initial correction. References: [12,29,16,33,32,34]. CI = confidence interval; RR = risk ratio

Correction: Six studies reported the rates of correction of the deformity for the two groups [12,16,29,32-34]. Four studies reported a significantly higher percentage of corrected feet in the Ponseti group compared to the control group [12,16,32,34]. Heterogeneity testing was non-significant (chi-square = 7.33, p = 0.198, I^2^ = 32%), so the fixed-effect model was used. The pooled RR [95% CI] was 1.23 [1.14, 1.32], with a p-value <0.001. Subgroup analysis showed a lack of significant difference (p = 0.137) (Figure 5). The leave-one-out analysis suggested that the study by Garcia et al. was influencing the results [33], as its omission reduced the I^2^ index to zero and slightly increased the pooled RR to 1.28 [95% CI = 1.19, 1.39].

**Figure 6:**
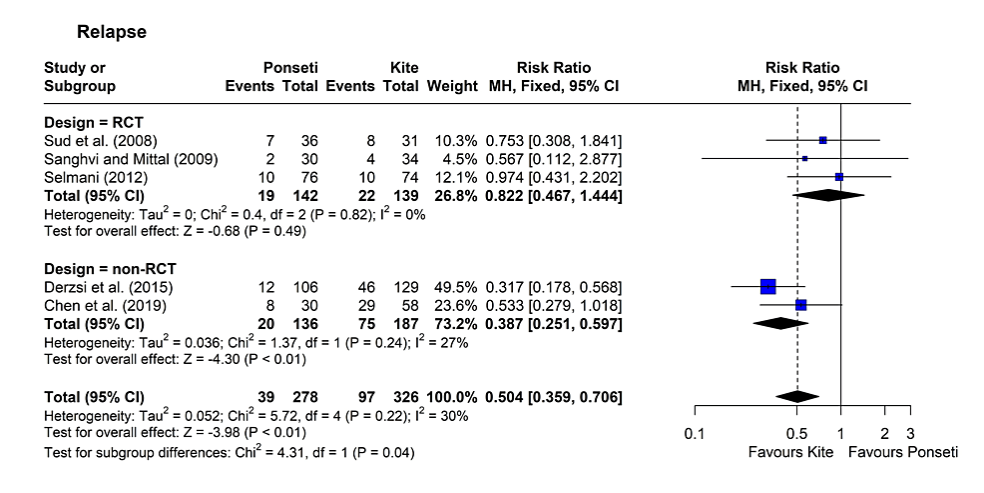
Forest plot showing pooling of the studies’ findings regarding the rate of relapse. References: [12,29,16,32,34]. CI = confidence interval; RR = risk ratio

Relapse: Five studies compared the rate of relapse after initial correction between the two arms [12,16,29,32,34]. All studies reported a lower rate of relapse in the Ponseti group compared to the Kite group, with statistical significance in two studies [32,34]. Heterogeneity was not significant (chi-square = 5.72, p = 0.221, I^2^ = 30%), so data were pooled using the fixed-effect model. The pooled RR [95% CI] was 0.50 [0.36, 0.71], with a p-value <0.001. Subgroup analysis showed a significant difference (p = 0.038), with lower RR in non-RCT studies (0.39 [95% CI = 0.25, 0.60]) compared to RCTs (0.82 [95% CI = 0.47, 1.44]) (Figure 6). The leave-one-out analysis showed that omitting the studies by Selmani and Derzsi et al. reduced heterogeneity (I^2^ index became zero) [16,32], though omitting the former reduced RR further (0.44 [95% CI = 0.30, 0.64]) while omitting the latter study increased the RR (0.69 [95% CI = 0.45, 1.05]).

Discussion

Summary of the Main Findings

Idiopathic clubfoot is one of the most common congenital deformities in children [2]. Currently, conservative methods are the preferred approach for initial treatment [7,8]. Several conservative techniques have been developed, with Kite and Ponseti techniques being the most commonly used [9,10]. The present meta-analysis aimed to compare the efficacy of the Kite and Ponseti methods in the treatment of children with idiopathic congenital clubfoot.

Nine studies met the eligibility criteria of this meta-analysis and were included. We found that the Ponseti method was significantly associated with a 23% probability of correction (n = 6, RR = 1.23 [95% CI = 1.14, 1.32], p < 0.001). The better success rate with the Ponseti technique may be attributed to the procedure of Achilles tendon tenotomy which is performed in selected patients [29]. Meanwhile, the difference in successful correction between the two techniques may also be the result of the non-correction of the heel deformity using the Kite method, which is commonly referred to in the literature as “Kite’s error” [9,29].

In addition, our results indicated that the Ponseti method was significantly associated with a lower risk of relapse by 50% (n = 5, RR = 0.50 [95% CI = 0.36, 0.71], p < 0.001) compared to the Kite method.

Ponseti explained the recurrence in idiopathic clubfoot by the continuation of excess collagen synthesis in the soft tissues of the lower limb rather than by the undercorrection of the deformity [5]. This is supported by the findings of Sud et al. who reported that most relapses in the Ponseti group showed all the components of clubfoot [12], suggesting the inadequacy of the use of force or non-compliance with maintaining the correct position after achieving correction. On the other hand, Sud et al. found that most relapses in the Kite group showed varus of the heel [12], either as an isolated deformity or combined with other components, suggesting a failure to achieve complete initial correction and that the relapsed cases were mislabelled as corrected.

Pooling of the studies’ findings showed that the Ponseti method required a lower number of casts (MD = -3.0 [95% CI = -5.8, -0.2], p = 0.04) and a shorter duration (MD = -39.76 [95% CI = -67.22, -12.30] days, p = 0.02) than the Kite method. The time for completing the casting in the Kite method typically ranges from four to eight months [9,10], as opposed to three to twelve weeks in the Ponseti method [12]. The higher number of casts and longer duration of treatment in the Kite method could be explained by the sequential correction of each component of deformity, while the Ponseti method simultaneously corrects all components except for the equinus deformity [29,32].

Previous meta-analyses compared between Kite and Ponseti methods and reported the superiority of the Ponseti method [40-43]. The review by Matos and de Oliveira included studies that used other conservative techniques in the control group besides Kite’s method [43]. The review by Gray et al. [41] included only one study by Rijal et al. in their meta-analysis [30]. Two of the previous meta-analyses addressed the evidence regarding the treatment of clubfoot in general, without focusing on the comparison between the Ponseti and Kite techniques [40,41]. The present meta-analysis includes more recent studies which were not included in the previous meta-analyses and focuses on the comparison between the two techniques.

Overall Completeness, Applicability, and Quality of the Evidence

The present meta-analysis shows that the Ponseti method is superior to the Kite method in terms of the higher rates of successful correction and lower rates of relapse. However, the included studies showed some limitations and potential ROB. The definitions and times for assessment of achieving correction were vague within the included studies. Only three studies used the Pirani score as an objective method for assessing correction. In addition, important data about the degrees of severity and age of initiating correction were not provided in most studies, despite their potential impact on affecting the outcomes. Another important limitation is the inadequate time for follow-up in some studies, as relapses may continue to appear until the age of four years [5].

Both the Ponseti and Kite methods possess advantages and disadvantages, which should be carefully considered when the method of correction is selected.

The advantage of the Kite method is that the casts can be changed every two weeks instead of weekly. This is an advantage in very poor communities where transport to the hospital and treatment costs may represent a barrier against continuing treatment. On the other hand, the time taken is much longer and the rates of residual deformity or relapse are higher than the Ponseti technique [9,10,33].

Meanwhile, the Ponseti method possesses the advantages of a shorter time of casting and better success rates. The drawbacks of the method include the longer cast which may introduce some difficulty while placing the cast and may lead to a higher rate of cast-related complications. Moreover, the casts need to be changed weekly, which may present a burden on families [29,30]. The impact of the socioeconomic status of the patients’ families was reported by Garcia et al. [33], as some patients were excluded from their study solely based on their inability to come to the hospital weekly. The same study had also excluded patients with allergies to the cast material.

## Conclusions

Evidence suggests that the Ponseti method results in better outcomes than the Kite method in terms of successful initial correction and lower relapse rates. The findings of this meta-analysis support the use of the Ponseti method over the Kite method for correction of idiopathic clubfoot. However, the available studies showed varying degrees of ROB, and the length of follow-up was inadequate in some studies.

## References

[1] Mustari MN, Faruk M, Bausat A, Fikry A (2022). Congenital talipes equinovarus: a literature review. Ann Med Surg (Lond).

[2] Smythe T, Kuper H, Macleod D, Foster A, Lavy C (2017). Birth prevalence of congenital talipes equinovarus in low- and middle-income countries: a systematic review and meta-analysis. Trop Med Int Health.

[3] Esbjörnsson AC, Johansson A, Andriesse H, Wallander H (2021). Epidemiology of clubfoot in Sweden from 2016 to 2019: a national register study. PLoS One.

[4] Pavone V, Chisari E, Vescio A, Lucenti L, Sessa G, Testa G (2018). The etiology of idiopathic congenital talipes equinovarus: a systematic review. J Orthop Surg Res.

[5] Ponseti IV (2002). Relapsing clubfoot: causes, prevention, and treatment. Iowa Orthop J.

[6] Khan SA, Kumar A (2010). Ponseti's manipulation in neglected clubfoot in children more than 7 years of age: a prospective evaluation of 25 feet with long-term follow-up. J Pediatr Orthop B.

[7] Besselaar AT, Sakkers RJ, Schuppers HA (2017). Guideline on the diagnosis and treatment of primary idiopathic clubfoot. Acta Orthop.

[8] Gelfer Y, Davis N, Blanco J (2022). Attaining a British consensus on managing idiopathic congenital talipes equinovarus up to walking age. Bone Joint J.

[9] Laaveg SJ, Ponseti IV (1980). Long-term results of treatment of congenital club foot. J Bone Joint Surg Am.

[10] Kite JH (1932). The treatment of congenital clubfeet: a study of the results in two hundred cases. JAMA.

[11] Kite JH (2003). Principles involved in the treatment of congenital club-foot. 1939. J Bone Joint Surg Am.

[12] Sud A, Tiwari A, Sharma D, Kapoor S (2008). Ponseti's vs. Kite's method in the treatment of clubfoot--a prospective randomised study. Int Orthop.

[13] Ponseti IV, Smoley EN (1963). Congenital club foot: the results of treatment. JBJS.

[14] Ponseti IV (1992). Treatment of congenital club foot. J Bone Joint Surg Am.

[15] Cooper DM, Dietz FR (1995). Treatment of idiopathic clubfoot. A thirty-year follow-up note. JBJS.

[16] Selmani E (2012). Is Ponseti's method superior to Kite's for clubfoot treatment he?. Eur Orthop Traumatol.

[17] Zhao D, Li H, Zhao L, Liu J, Wu Z, Jin F (2014). Results of clubfoot management using the Ponseti method: do the details matter? A systematic review. Clin Orthop Relat Res.

[18] Liberati A, Altman DG, Tetzlaff J (2009). The PRISMA statement for reporting systematic reviews and meta-analyses of studies that evaluate health care interventions: explanation and elaboration. Ann Intern Med.

[19] Sterne JA, Savović J, Page MJ (2019). RoB 2: a revised tool for assessing risk of bias in randomised trials. BMJ.

[20] Slim K, Nini E, Forestier D, Kwiatkowski F, Panis Y, Chipponi J (2003). Methodological index for non-randomized studies (minors): development and validation of a new instrument. ANZ J Surg.

[21] Kim SY, Park JE, Lee YJ (2013). Testing a tool for assessing the risk of bias for nonrandomized studies showed moderate reliability and promising validity. J Clin Epidemiol.

[22] (2021). R Core Team. R: A Language and Environment for Statistical Computing. Vienna, Austria: R Foundation for Statistical Computing. https://www.scirp.org/reference/referencespapers?referenceid=3456808.

[23] Balduzzi S, Rücker G, Schwarzer G (2019). How to perform a meta-analysis with R: a practical tutorial. Evid Based Ment Health.

[24] Harrer M, Cuijpers P, Furukawa T (2019). Doing Meta-Analysis With R: A Hands-On Guide. https://www.routledge.com/Doing-Meta-Analysis-with-R-A-Hands-On-Guide/Harrer-Cuijpers-Furukawa-Ebert/p/book/9780367610074.

[25] Higgins JP, Thompson SG, Deeks JJ, Altman DG (2003). Measuring inconsistency in meta-analyses. BMJ.

[26] Segev E, Keret D, Lokiec F, Yavor A, Wientroub S, Ezra E, Hayek S (2005). Early experience with the Ponseti method for the treatment of congenital idiopathic clubfoot. Isr Med Assoc J.

[27] Shyam AK (2011). Comparison of Ponseti and Kite's method of treatment for idiopathic clubfoot. Indian J Orthop.

[28] Primadhi A, Ismiarto YD (2009). Comparison between Kite-Lovell method and Ponseti in clubfoot treatment. Bandung Med J.

[29] Sanghvi AV, Mittal VK (2009). Conservative management of idiopathic clubfoot: Kite versus Ponseti method. J Orthop Surg (Hong Kong).

[30] Rijal R, Shrestha BP, Singh GK, Singh M, Nepal P, Khanal GP, Rai P (2010). Comparison of Ponseti and Kite's method of treatment for idiopathic clubfoot. Indian J Orthop.

[31] Kaseke F, Mudawarima T (2013). Comparison of Ponseti and Kite's method of treatment for congenital Talipes Equino using the Pirani scoring system. Cent Afr J Med.

[32] Derzsi Z, Nagy Ö, Gozar H, Gurzu S, Pop TS (2015). Kite versus Ponseti method in the treatment of 235 feet with idiopathic clubfoot: results of a single Romanian medical center. Medicine (Baltimore).

[33] Garcia LC, de Jesus LR, Trindade MO, Garcia FC, Pinheiro ML, de Sá RJ (2018). Evaluation of Kite and Ponseti methods in the treatment of idiopathic congenital clubfoot. Acta Ortop Bras.

[34] Chen C, Wang TM, Wu KW, Huang SC, Kuo KN (2019). Comparison of two methods for idiopathic clubfoot treatment: a case-controlled study in Taiwan. J Formos Med Assoc.

[35] Ippolito E, Farsetti P, Caterini R, Tudisco C (2003). Long-term comparative results in patients with congenital clubfoot treated with two different protocols. J Bone Joint Surg Am.

[36] Herzenberg JE, Radler C, Bor N (2002). Ponseti versus traditional methods of casting for idiopathic clubfoot. J Pediatr Orthop.

[37] Smith PA, Kuo KN, Graf AN (2014). Long-term results of comprehensive clubfoot release versus the Ponseti method: which is better?. Clin Orthop Relat Res.

[38] Garhwal P, Choudhary BJ (2018). Assessment of efficacy of Ponseti and Kite’s method of treatment for idiopathic clubfoot: a comparative study. Indian J Basic Appl Med Res.

[39] Sharif S, Khan RR, Riaz S (2021). Effectiveness of Ponseti versus Kite method for the management of club foot-a quasi experimental trial. Pak J Med Health Sci.

[40] Bina S, Pacey V, Barnes EH, Burns J, Gray K (2020). Interventions for congenital talipes equinovarus (clubfoot). Cochrane Database Syst Rev.

[41] Gray K, Pacey V, Gibbons P, Little D, Burns J (2014). Interventions for congenital talipes equinovarus (clubfoot). Cochrane Database Syst Rev.

[42] He JP, Shao JF, Hao Y (2017). Comparison of different conservative treatments for idiopathic clubfoot: Ponseti's versus non-Ponseti's methods. J Int Med Res.

[43] Matos MA, de Oliveira LA (2010). Comparison between Ponseti's and Kite's clubfoot treatment methods: a meta-analysis. J Foot Ankle Surg.

